# An Evaluation of Understandability of Patient Journey Models in Mental Health

**DOI:** 10.2196/humanfactors.5640

**Published:** 2016-07-28

**Authors:** Jennifer Percival, Carolyn McGregor

**Affiliations:** ^1^ University of Ontario Institute of Technology Faculty of Business and Information Technology Oshawa, ON Canada

**Keywords:** patient-journey modeling, process modeling, technology integration, health information technology

## Abstract

**Background:**

There is a significant trend toward implementing health information technology to reduce administrative costs and improve patient care. Unfortunately, little awareness exists of the challenges of integrating information systems with existing clinical practice. The systematic integration of clinical processes with information system and health information technology can benefit the patients, staff, and the delivery of care.

**Objectives:**

This paper presents a comparison of the degree of understandability of patient journey models. In particular, the authors demonstrate the value of a relatively new patient journey modeling technique called the Patient Journey Modeling Architecture (PaJMa) when compared with traditional manufacturing based process modeling tools. The paper also presents results from a small pilot case study that compared the usability of 5 modeling approaches in a mental health care environment.

**Method:**

Five business process modeling techniques were used to represent a selected patient journey. A mix of both qualitative and quantitative methods was used to evaluate these models. Techniques included a focus group and survey to measure usability of the various models.

**Results:**

The preliminary evaluation of the usability of the 5 modeling techniques has shown increased staff understanding of the representation of their processes and activities when presented with the models. Improved individual role identification throughout the models was also observed. The extended version of the PaJMa methodology provided the most clarity of information flows for clinicians.

**Conclusions:**

The extended version of PaJMa provided a significant improvement in the ease of interpretation for clinicians and increased the engagement with the modeling process. The use of color and its effectiveness in distinguishing the representation of roles was a key feature of the framework not present in other modeling approaches. Future research should focus on extending the pilot case study to a more diversified group of clinicians and health care support workers.

## Introduction

### Health Information Technology Prospects

Health information technology (HIT) is expected to improve patient care through increased accessibility to high-quality information, reduction in documentation efforts, and general overall time savings for clinicians [1]. For these reasons, there have been numerous initiatives to spur investment in HIT including computerized order entry systems, electronic medical records (EMRs), and more complex clinical decision support systems [2-4]. Governments, hospitals, clinics, and individual physicians have been investing millions of dollars into HIT. This is a large investment for both the government and physicians, especially given the lack of confidence that the implementation of EMR will result in a positive return expressed by many physicians [2]. Various studies [5-9] have proven that the advances in health care, especially HIT, are not being incorporated by practitioners into clinical best practices. Recent studies have also focused on identifying the unintended consequences of HIT implementations [10-17], and in particular, the importance of the effects of organizational constraints on HIT remains an understudied domain [18]. The implementations of HIT have been predicated not only by monetary and fiscal constraints but also by other organizational factors as well such as access to innovative technologies, the applicability of the HIT to clinical practice, and the attitudes of the clinicians themselves [19,20].

Although technical barriers and system design flaws do exist, these are too often the source for blame when HIT implementation failures or undesirable consequences arise [21,22]. Many of the undesirable consequences are a result of human and sociotechnical interactions (the interactions between new HIT and the organization’s culture), including in particular their workflows, team dynamics, communications structures, and existing information systems [17,23]. Due to the increased demand for demonstrating meaningful use and integration of HIT into clinical practice, changing the current methods for evaluating the integrated potential of HIT is critical for all health care organizations [24,25]. Kaplan [26] found that one of the primary barriers in the managing of HIT design and implementation projects was communication and understanding of the workflow-related issues stemming from the broad spectrum of stakeholders involved in the projects: “Participants described the difficulty in fully understanding workflow, as evidenced by the workflow changes resulting in endless workarounds.” We propose the use of patient journey models to provide a clear visual representation of the workflows, technology, and communication interactions. Using visual models to depict health care situations enables all stakeholders to audit current practices and subsequently strategically plan process improvement initiatives focused on patient safety, quality of care, and efficiency [27].

Many studies on HIT evaluation methods support the need for improved modeling techniques to meet the specific complexities and social contexts of health care [28,29]. The modeling of information flows and integration into practice in HIT evaluation studies continues to be an issue requiring additional research. Process modeling has traditionally been used to improve information flows within organizations [27,30]. These techniques use basic flow charts [31], lean process mapping, or other methods derived from the manufacturing sector [9,32-34]. Recently, work has focused on modeling processes through the lens of the patient using various patient journey modeling (PJM) techniques [27,35]. These models can help both administrators and clinicians understand potential consequences of changes in processes and information flows due to HIT implementations. Using these models as a component of existing HIT evaluation methods, it will be possible to determine a set of unique clinical care processes based on the organization’s culture that integrate EMR systems for the benefit of improved patient care.

Although various modeling techniques are being used in support of quality improvement and technology adoption, there remains an issue of whether those affected by the organizational change are able to assess the potential impact based on how the information is represented. In our earlier research [36], we have demonstrated the difference when 2 modeling techniques are used to represent the same patient journey from a functional matrix perspective. This paper presents a comparison of 5 process modeling techniques with a focus on supporting HIT integration into clinical practice. The results of an initial pilot study of user perceptions of the understandability of the representation of a patient journey model within which they actively participate across 5 process mapping techniques is also presented to provide support for the theoretical constructs. This research was part of a larger EMR technology adoption change management initiative at Providence Mental Health Care, Kingston, Ontario.

### Background

Given the current era of technology development, there are a number of research findings that support the utilization of advancing HIT in clinical practice [2,9,37]. Although the benefits of using HIT in the health care setting have been proven to improve patient care, “adapting new information systems to health care has proven difficult, and rates of use have been limited” [38]. There are many HIT resources available, such as EMR, computerized physician order entry systems, and clinical decision support systems that enable improved patient care through timely delivery of secured patient information. However, a number of studies have identified unintended consequence to workflows as a major issue in HIT implementations [10-13,15,16]. We refer the reader to Greenhalgh’s systematic review summarizing the tensions and paradoxes in EMR research results for a synopsis of this work [39].

A number of studies have examined nurses’ perceptions of EMR, and more generally HIT, to understand the barriers to technology integration in health care [40-43]. These studies have found that although nurses are open to the possible benefits of EMR and HIT, they continue to have concerns about how these technologies will integrate into bedside care. Results from studies on physicians using EMR have supported similar concerns [11,39,44,45]. Many technology adoption–led change management initiatives failed to enable people in various health care roles to fully understand their future work practice behaviors. In their systematic review of HIT implementations, Cresswell and Sheikh [18] found that the implementation of HIT has been noted to have significant challenges in integrating a range of interrelated technical, social, and organizational factors necessary to fully integrate the technology with clinical practice. These challenges present opportunities for the utilization of a process modeling architecture that integrates technical, social, and organizational factors into the process modeling to convey information effectively and enabling both HIT designers and clinicians to clearly understand the proposed future work practices.

To improve patient safety and quality under increasing budget constraints, researchers in health management began to modify business process modeling techniques from traditional manufacturing applications [9,32]. Recently, a number of studies have looked at lean approaches for process re-engineering and cost reductions [46-50]. A significant amount of research has focused on a patient-focused model to analyze problems occurring in health care [6,7,51]. Identification of such “system of care” improvements is the primary objective of PJM initiatives through a patient-centric activity that details a patient’s progress through a health care system for a given service [52]. PJM aims to improve patient safety and overall health care quality by highlighting patient information flow issues and thereby aiding in the reduction of variability in the care process. The results of the analysis, combined with the provider goals, are used to derive target processes and justify change management proposals [53,54]. Creating clinical care pathway models that focus on the patient’s perspective aid in the identification of potential unintended consequences of HIT implementations, as well as potential innovations related to the use of HIT at all levels of the organization. Clearly, presented models aid in identifying gaps or inefficiencies in information flow, workflows that integrate EMR, and providing visual representations of clinical practice for improved consistency in quality of care. Improving the understanding of the sociotechnical issues will facilitate communication between stakeholders. It will also increase the level of understanding of the potential consequences to workflow and communication patterns due to the HIT implementation [12,13]. Unfortunately, gaining this insight continues to become more challenging as the technological and institutional changes in health care increase the complexity of the workflows and related social interactions. These social interactions continue to be difficult to integrate within many modeling techniques [43].

Modeling of the multiple dimensions that contribute to the entire journey experienced by a patient within and across hospitals, clinics, and community health organization(s) must include the inherent complexity of their inter-relationship that influence the structure, processes, and outcomes of the service system [55,56]. Therefore, from a high-level perspective, the process of PJM is to optimize improvement of services and innovation across structural changes, process improvements, and outcome improvements simultaneously. At a more specific level, PJM provides direct opportunities for improvements and process innovation in areas such as improved information flows among all members of the health care team including the patient and their family, streamlined handovers between and across health care organizations, elimination of duplicated work and data collection, and increased compliance to organizational policies. The use of PJM also increases the level of engagement and empowerment of employees and patients through their involvement in the modeling process. The results of the analysis of the patient journey models, combined with the provider goals, are used to derive future desired processes and justify change management proposals [55,56]. By analyzing the models (both those representing the current state and those predicting the future, post-HIT implementation state), designers of HIT systems as well as practitioners can better understand the sociotechnical limitations of the organization. This is important, as it will help identify potential consequences to the clinical and administrative processes mediated by the HIT implementation. Given the complexity of the collaborative work and multiple information flows among people, information systems, documents, and organizational processes [57,58], these models provide a comprehensive view of how changes in HIT will affect existing paradigms. However, if the PJM created does not correctly reflect the current state and this is not detected by staff due to issues of model understandability, then those unrepresented activities are not included within the quality improvement or technology adoption initiative. This has great potential to lead to issues with future state implementation.

There are also a number of limitations to existing patient journey and process modeling techniques [59]. Existing models have limitations on the details that can be represented in them. There is also concern about the usability of developed models [60]. Most modeling methodologies have specific languages developed from information systems and have not been developed with novice modelers or involvement of the general public in mind [61]. The modeling methodologies each have a unique notation, which does not leverage aspects of perceptual discriminability and semantic transparency [62]. These languages are difficult for most people in an organization to understand and therefore limit the number of employees who can easily be engaged in the modeling process as well as the clarity of the developed models. To develop and easily maintain process models, it is important to engage employees at all levels, as this will significantly increase the organization’s ability to identify possible innovation opportunities and improve the efficient and effectiveness of patient care.

Recently, there has been a growing interest in using visual process models for communication and as a tool to support change management initiatives in organizations [63]. Process models are also being considered for use in staff training, customer or patient information, or as teaching tools in higher education [64]. These applications require a model that is intuitive, clear, and easily understood. The models must also be comprehensive to ensure a high degree of knowledge transfer. To achieve these outcomes, the process modeling notation must exhibit a high degree of cognitive effectiveness [62]. It is the involvement of the stakeholders that supports change management and the development of lasting process innovations as they become aware of inefficiencies through the visual analysis of the process models as they are developed and refined [65].

The health care environment is also quite different from manufacturing or even many other services. In particular, unlike most lean initiatives where duplication should always be eliminated, in health care, some duplication is essential for patient safety and specified in clinical protocols (eg, medication reconciliation at all handovers) [65]. Health care also tends to have a greater number of decision points due to the complexity of comorbidities. These result in data being recombined in a number of ways to support decision making throughout the patient journey. The decision-making process typically integrates a coordinated team approach making many process modeling approaches unable to adequately capture the team dynamics and role differentiation [66]. Health care also has a high level of required documentation. This increases the need to represent in the process models how information is recorded and the standardization of this data collection [67]. Finally, it is critical that health care process models include policies and guidelines that support each process step. Capturing these data sources is critical for identifying potential process improvements and areas where HIT could be leveraged for compliance with best practice approaches [65].

**Figure 1:**
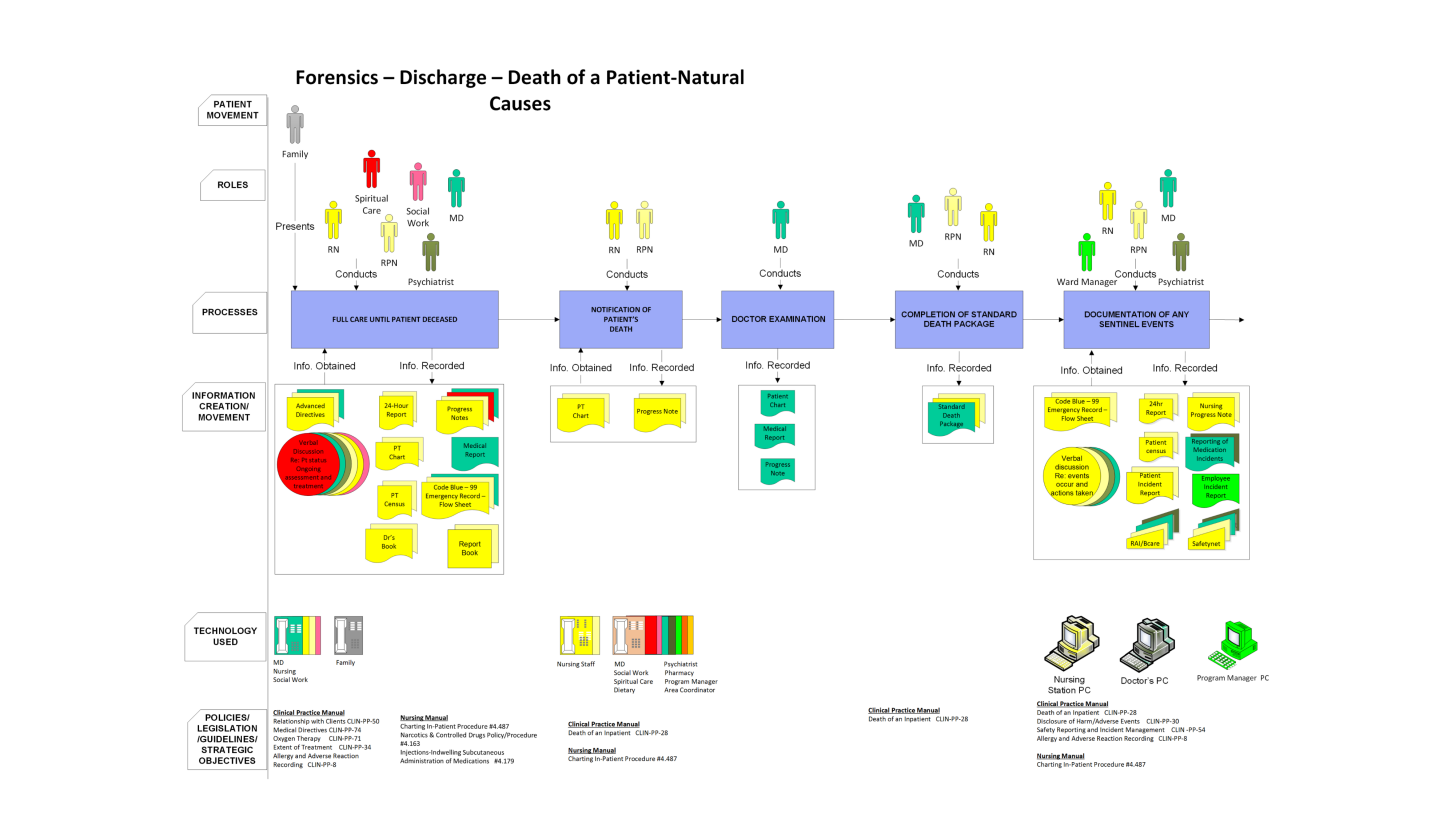
PaJMa Model of Forensic Unit Discharge Process.

The Patient Journey Modeling Architecture (PaJMa) is a patient journey modeling methodology that enables a visual representation of the interaction of processes, technologies, and people used to support a patient’s experience in the health care system [9,36]. This modeling technique represents the following layers: staff roles, processes, information creation/movement, HIT, IT infrastructure, patient needs/practice guidelines/policies, and metrics [51] (refer to Figure 1 for an example). This ensures the visual integration of all the major elements in Sittig and Singh’s [68] sociotechnical model for studying HIT in complex health care environments. The updated architecture also uses color coding to aid in the identification of which role is the primary user of the information source and those that are also subusers of information recorded in the information source. Color is a powerful visualization means allowing for the identification of different or similar roles and processes [69]. The use of color supports redundant coding and has been shown to reduce noise and protect the transfer of information from interpretation errors [62]. This color coding helps in the requirements gathering process by clearly identifying the roles that require access to the various set of information and at what stage in the patient journey this information is recorded, updated, or simply accessed to support decision making. Similarly, to aid in the identification of the number and types of technology resources (both input and output devices) that are required, as well as infrastructure needs, these too are color coded to indicate the individuals who use the devices. The networks that are used, whether internal to the hospital, links to external care providers, or patient homes are also color coded to aid in the specification of any security and/or infrastructure needs that would have to be considered if the process were to be altered. This is particularly important when considering many of the new eHealth initiatives to support home-based care or self-monitoring of chronic conditions.

The use of the PaJMa approach aids in visually depicting the current care processes within a particular health care unit or facility as well as the potential future state after HIT implementations. PaJMa is an effective method for pointing out inefficiencies and allowing health care professionals to work with and alter the model to benefit their practices [36]. The PaJMa model is the only model that integrates IT into the representation while enabling the explicit representation of the guidelines and/or protocols that relate to tasks within the process model and the only approach that supports patient needs.

## Methods

### Comparing the PJM Methods

In Table 1, we present a comparison of key aspects of process definition required for PJM. This comparison is not meant to provide a complete functional comparison from a business process perspective, but rather to highlight some key requirements within the domain of health care.

**Table 1:** Comparison of patient journey modeling techniques.

Description	Data flow diagram	Flow chart	IDEF-0	Lean VSM	PaJMa
**Process definition**					
Definition of tasks	Yes	Yes	Yes	Yes	Yes
Decompose tasks to subtasks	Yes	Yes	Yes	Yes	Yes
Construct process model	Yes	Yes	Yes	Yes	Yes
Conditional paths	Implicit	Explicit	Implicit	Explicit	Explicit
Expected task times	No	No	No	Yes	Yes
Expected queue times	No	No	No	No	Yes
**Roles**					
Definition of roles	Yes	Sometimes	Yes	Yes	Yes
Roles to process definition	Yes	Sometimes	Yes	Yes	Yes
Roles to information	Explicit	No	Implicit	Implicit	Explicit
**Information**					
Information storage name	Yes	No	Yes	Yes	Yes
Information storage medium	No	No	No	Yes	Yes
Information access technology	No	No	No	No	*Yes*
Information network access	No	No	No	No	*Yes*
Information creation	Implicit	Implicit	Implicit	Implicit	Explicit
Information retrieval	Implicit	Implicit	Explicit	Implicit	Explicit
**Guidelines and protocols**					
Guideline associated with task	No	Sometimes	No	No	Yes
**Patient needs**					
Cultural needs associated with tasks, eg, interpreter	No	No	No	No	Yes
Religious needs associated with tasks, eg, female patient not left alone with a male health care practitioner	No	No	No	No	Yes
**Metrics**					
Expected task times	No	No	No	Yes	Yes
Expected queue times	No	No	No	Yes	Yes
Task cost	No	No	No	Yes	Yes
Task targets	No	No	No	Yes	Yes

The comparison is grouped based on process definition, roles, information, guidelines and protocols, patient needs, and metrics. This comparison supports the recent trend for the use of Lean Value Stream Mapping [70,71] as it shows the functional quality of the approach. However, the PaJMa model is the only model that enables the explicit representation of the guidelines and/or protocols that relate to tasks within the process model and the only approach that supports patient needs [72]. It is also the only model that integrates the technical aspects of the information systems infrastructure along with the data requirements.

Understanding the benefits that EMR can bring to the patient, health care team, and to the delivery of care is an important part of systems implementation planning. The use of patient journey models has been shown to be very beneficial in the systems requirement gathering process as it combines the perspectives and needs of all members of the health care team into a cohesive vision [36]. These diagrams are also extremely valuable to the systems development team for identifying the upgrade possibilities with the highest impact on patient care, in supporting change management initiatives, and improving user support for the EMR system [73]. Once the benefits of EMR implementation have been analyzed, the developers and health care providers must then integrate the use of EMR to clinical practice and minimize the potential for unintended consequences.

### Case Study

To explore and validate the understandability of the PJM frameworks, we used a qualitative/quantitative mixed methods approach with 17 health care practitioners from the Forensics Ward and Adult Rehabilitation Ward at Providence Mental Health Care in Kingston, Ontario. The participants consisted of the entire clinical team working on the electronic patient record initiative for the design of the organization’s EMR system. The study was approved by the University of Ontario Institute of Technology research ethics board and was run at the host site as the first project under a memorandum of understanding to support the University in its teaching and research with undergraduate and graduate students in health sciences and health informatics. This is a small pilot study to support the conceptual model developed, and all results should be viewed with an understanding of this limitation.

A brief introduction to process modeling was presented to participants to give them a little background with regard to the purpose of the research and the survey instrument. The survey instrument explores 3 key aspects of the model architectures: (1) personal factors and model factors which affect the reader`s understandability of the model; (2) whether these models are sufficient for clinician understanding; and (3) the comparison between various modalities of models. The survey instrument is available from the authors on request.

A process used on the participants’ unit was modeled, and models using 5 different modeling techniques were presented; data flow diagram, IDEF-0, traditional flowchart, lean, and PaJMa model. Once all 5 models were presented, the models remained visible to the participants and a survey was then conducted to collect feedback on preferences. These different models were presented to compare and contrast the differing PJM frameworks in terms of ease of understanding the process, ease of identifying their own role within the model, and overall visual aspects of the models. None of the participants had used the PaJMa or other frameworks before in their work processes, although many were familiar with flow charts.

**Figure 2:**
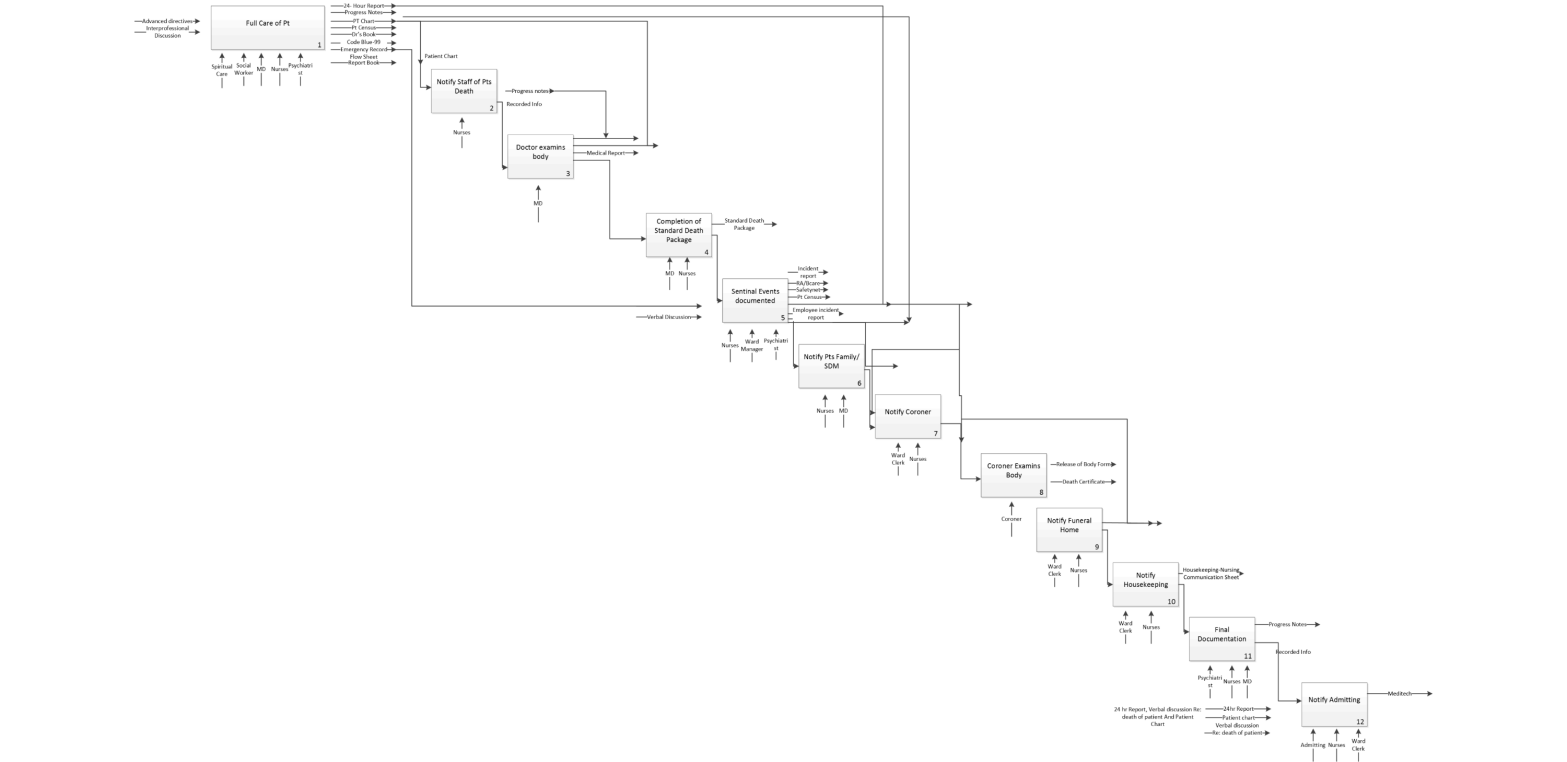
IDEF-0 Model of Forensic Unit Discharge Process.

**Figure 3:**
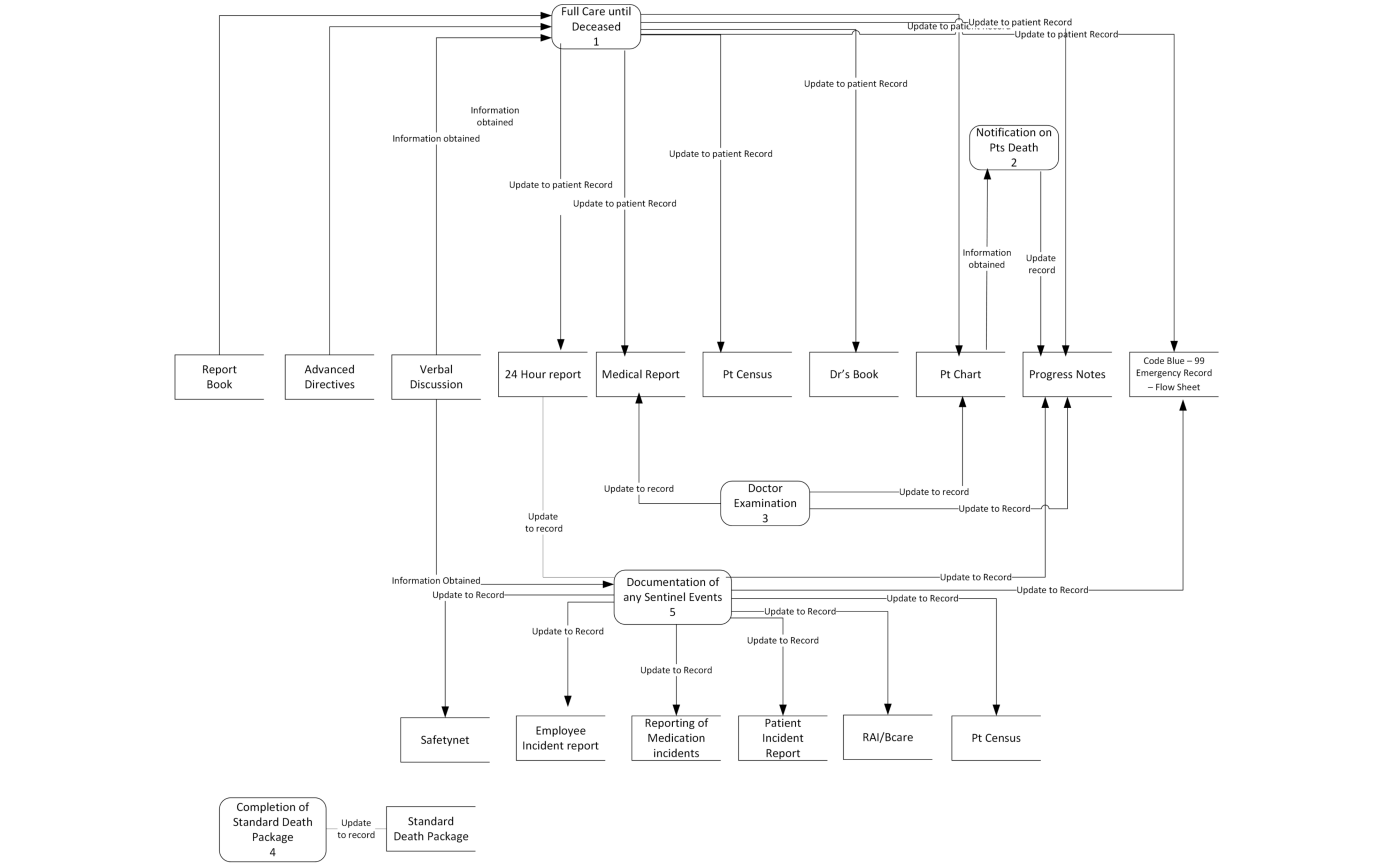
Data Flow Diagram of Forensic Unit Discharge Process.

**Figure 4:**
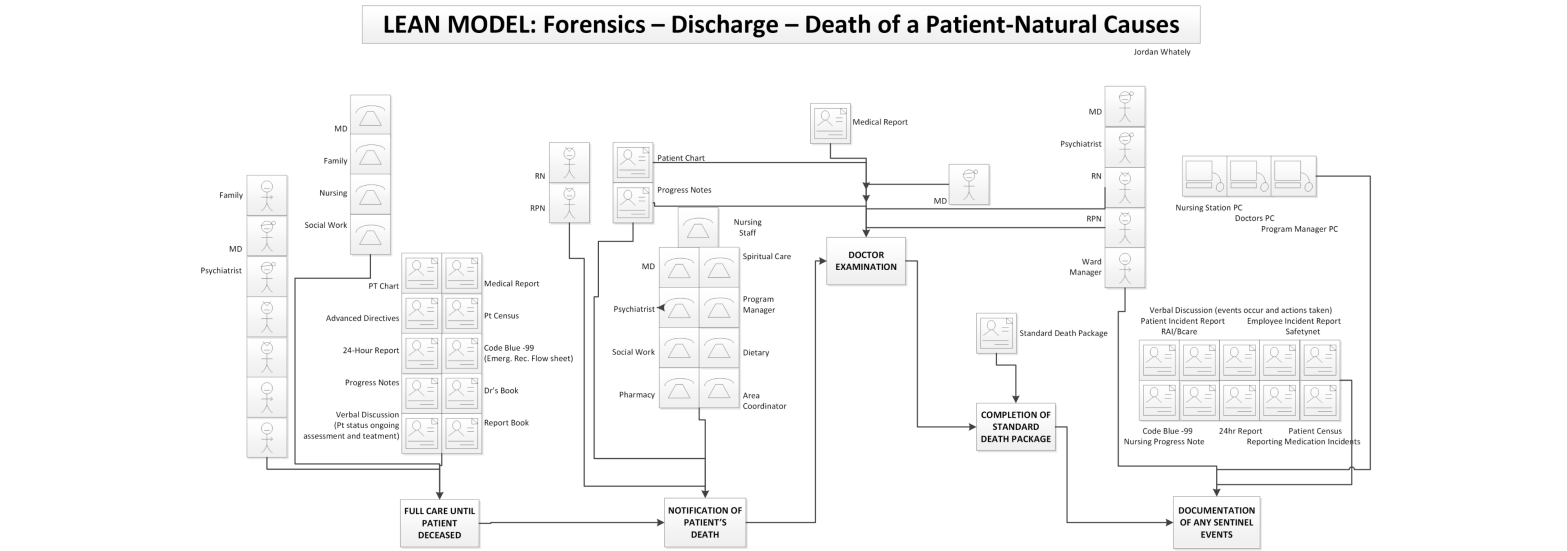
Lean Value Stream Map of Forensic Unit Discharge Process.

Figures 1-4 present a matching segment of the larger process used as part of the study due to space restrictions. The standard flow chart example was not included in the paper but is available from the authors on request. Although only showing a segment, it still illustrates the functionality of each modeling technique for the purposes of reporting our research findings. The IDEF-0 (refer to Figure 2) and data flow diagram (refer to Figure 3) are techniques derived from information systems research. These techniques focus on supporting systems development but are not intuitive for novice patient journey modelers. The techniques were found to be difficult for care providers to understand, and they were found to have limited ability to incorporate key elements in understanding the patient journey such as policies, guidelines, and caregiver roles.

The participants were also asked to explain how the use of color affected their overall ranking on the models. They were then asked to focus on the PaJMa model and were provided with the same model in color and black and white to explore how color affected the perceptions on the usability of this modeling method. Participants were given the opportunity to express their rationale for selection of preferred modeling methods as well as asked to provide specific aspects of the modeling framework that contributed to ease of understanding and improved organization of the data regarding information flow in the patient journey. Figure 3 represents an example of the PaJMa model presented and the same process is mapped as a Lean Value Stream Map in Figure 4.

## Results

**Figure 5:**
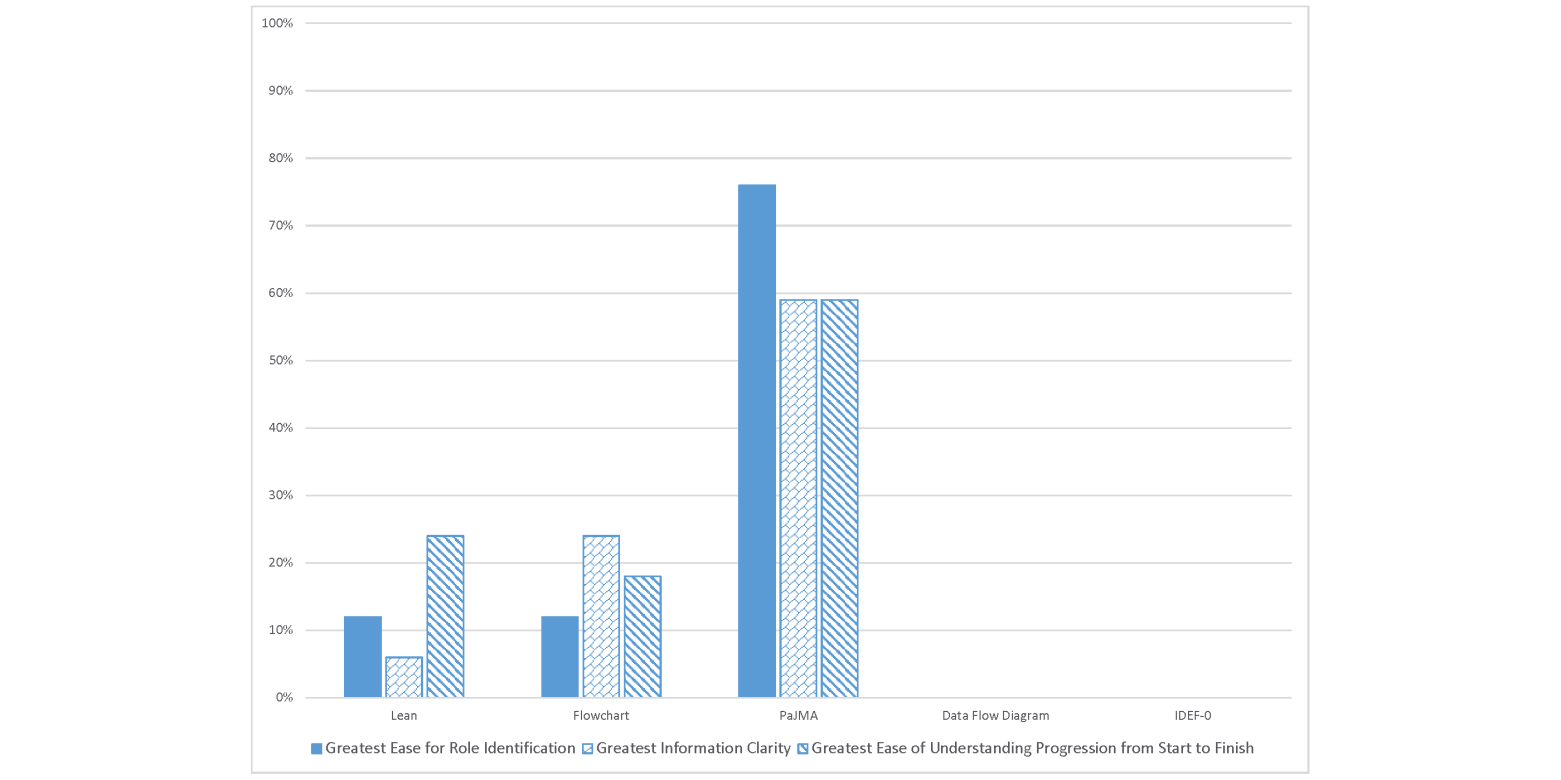
Comparison of Ease of Use of Patient Journey Models.

The results from the survey suggest that the health care professionals understood the PaJMa model more easily than the Lean Value Stream Map that was presented. From the data, 7 (41%) of participants found the PaJMa model to be the most visually pleasing when compared with the other models with a basic flow chart next at 4 (24%) and a Lean Value Stream Map next at only 3 (18%). When we focused the analysis on role identification, information clarity, and general ease of flow progressing through the model, respondents found the PaJMa model to outperform the other options (Figure 5).

When asked about what would be important factors for using the models, 13 (76%) of participants considered the length of time working on the ward, the amount of experience with models, or both factors together as key characteristics for determining how a staff member may interpret the models being used on the wards. In addition to this, when participants were asked about factors of the model that they noticed contributed to the enhancement of the model’s usability, 13 (76%) of participants mentioned the use of color in their answers. It was found that 14 (82%) of the participants favored the PaJMa model with its use of color compared with the same black and white version of the PaJMa model. Statements made included: “the colour, pictorial diagrams, layers, and explanations linked (shape and color) contributed to the usability of the model. The presentation/explanation on how to read the model and inclusion of staff was great” and “size, color, role representation, and shapes of various items used on the models contribute to the ease of interpretation.” This demonstrates that color is an important, and easily implemented, element that should be leveraged in all modeling methodologies. The results support the conclusion that the PaJMa approach has increased clarity and overall cognitive effectiveness than the other models.

## Discussion

### Principal Findings

Although workflow models must encompass accurate details of the processes it illustrates, it must be able to do so in a way that allows each piece of the puzzle to be distinct and discernible from one another. We found that those who participated in the survey favored the characteristics of the PaJMa model such as color, size, and the structured approach of the layout. The PaJMa model allows for current processes to be laid out as they presently are, and feedback from the stakeholders will be used to update these models to reflect the thoughts of all the stakeholders. While these models provide valuable insight into potential consequences of HIT implementations, these insights are limited by the accuracy of the models. Models that detail the current and future HIT-enabled processes, taking into account the opinions and feedback of a variety of stakeholders, are valuable tools in the design and implementation of HIT systems and eHealth services. The high level of usability and access by front-line practitioners will ensure increased adoption of the model and will support the minimization of errors, ultimately improving the understanding of all stakeholders and improving the quality of patient care.

The use of the PaJMa framework will enable health care organizations to clearly visualize how EMR, and HIT in general, can be beneficial for themselves and their patients. By developing their own unique sets of models, each organization will gain greater depth of understanding on their sociotechnical constraints including the requirements that their organizational culture and practices have for an EMR implementation. The use of this type of modeling will also support a more effective and easier implementation of HIT, as health care professionals can visualize the benefits and challenges before implementing the new technology. This will allow new practices to be developed and training of all staff to take place before the new system is implemented. The models can also serve as a process measurement tool enabling improved analysis of the benefits obtained once the implementation is complete.

### Conclusions

This paper has presented preliminary assessment of the understandability of the PaJMa framework to aid in effectively integrating HIT into clinical practice through visualization of current and future patient journeys. The incorporation of EMR into clinical practices is essential to the future of health care. Not only will it increase accessibility to patient information but also will increase patient safety, support patient confidentiality, and decrease time spent reviewing and asking about a patient’s health history thereby improving patient care and the sustainability of the health care system. These models help improve practitioner and HIT designer’s understanding of the network of information flows and cultural relationships that shape the organization’s workflow patterns. Understanding these elements has been linked [20,74] to mitigating unintended consequences of such HIT implementations.

This research demonstrated increased staff understandability of the representation of their processes and activities within the PaJMa models and higher degrees of engagement in the change process [75]. The results indicated that the modeling approach was valuable to the host organization and was of interest to the consulting company working on the development of the electronic patient record project. The PaJMa methodology is also currently being utilized as part of a HIT capacity audit across Canadian Neonatal Intensive Care Units (NICUs). Future research will look into how to adequately transform an organization to use new practice guidelines that integrate HIT and how to leverage patient journey models to support improved EMR design.

## Abbreviations

EMR: electronic medical record
HIT: health information technology
NICU: Neonatal Intensive Care Unit
PaJMa: Patient Journey Modeling Architecture
